# Mucosal Vaccination with Recombinant Adenovirus Encoding Nucleoprotein Provides Potent Protection against Influenza Virus Infection

**DOI:** 10.1371/journal.pone.0075460

**Published:** 2013-09-25

**Authors:** So-Hee Kim, Joo Young Kim, Youngjoo Choi, Huan H. Nguyen, Man Ki Song, Jun Chang

**Affiliations:** 1 Graduate School of Pharmaceutical Sciences, Ewha Womans University, Seoul, Korea; 2 Laboratory Science Division, International Vaccine Institute, Seoul, Korea; KAIST, Graduate School of Medical Science & Engineering, Republic of Korea

## Abstract

Influenza vaccines that target the highly variable surface glycoproteins hemagglutinin and neuraminidase cause inconvenience of having vaccination every year. For this reason, development of universal vaccines targeting conserved viral components is needed. In this study, we generated recombinant adenovirus (rAd) vaccine encoding nucleoprotein (NP) of A/PR/8/34 influenza virus, designated rAd/NP. BALB/c mice were immunized intranasally or sublingually with rAd/NP vaccine and subsequently challenged with lethal doses of heterologous as well as homologous influenza viruses. We found that intranasal immunization of rAd/NP elicited strong mucosal IgA responses as well as stronger CD8 T-cell responses toward immunodominant K^d^-restricted NP_147-155_ epitope than sublingual immunization. Importantly, only single intranasal but not sublingual immunization of rAd/NP provides potent protection against both homologous and heterologous influenza virus challenges. These results suggest that recombinant rAd/NP could be a universal vaccine candidate for mucosal administration against influenza virus.

## Introduction

Influenza virus is an important respiratory pathogen accounting for 3-5 million infections and responsible for 250,000-500,000 deaths annually worldwide [1]. Recently, newly emerging influenza virus subtypes have infected humans and caused significant public health concerns. For example, since several human cases of highly pathogenic H5N1 avian influenza virus infection have been first reported in Hong Kong in the late 1990s, hundreds of additional confirmed cases of human infection by H5N1 virus have been reported with a lethal outcome in over 50% of the documented cases [2,3,4]. Also, in 2009, a new swine/human/avian-origin H1N1 influenza virus emerged in Mexico and resulted in a worldwide pandemic [5]. Moreover, recent outbreak of H7N9 avian influenza virus in China has claimed multiple human lives, while the numbers of reported human cases are growing continually [4]. Hence, these examples underscore the necessity for better preparedness against potential influenza virus pandemic caused by different influenza virus strains.

Vaccination is the most cost-effective way to control and/or prevent influenza outbreaks. However, live-attenuated and inactivated influenza vaccines that are currently licensed for human use are designed to induce strain-specific humoral immunity and cannot offer cross-protection against different strains of influenza virus expressing sequentially and/or conformationally related, but unique, viral surface proteins generated by random antigenic changes that influenza virus frequently undergo. Thus, development of vaccines that offer broad-range protection against multiple strains of influenza virus can be immensely beneficial for public health. For development of such influenza vaccines, it is important to consider that the immune response elicited by the vaccination targets viral antigens that are highly conserved among multiple influenza virus strains.

Influenza virus nucleoprotein (NP) contains a conserved immunodominant CD8 T-cell epitope which is associated with the induction of cross-protective immunity against heterologous and heterosubtypic influenza virus infections [6,7,8]. It has been previously shown that immunization with recombinant adenovirus (rAd) vaccines encoding conserved influenza antigens such as NP and M2e generated cross-reactive immune responses, which can provide protection from lethal virus challenge in mice [9,10,11]. Accordingly, in our present study, we generated a recombinant adenovirus expressing full-length NP (rAd/NP) derived from influenza virus A/Puerto Rico/8/1934 (PR8) and evaluated its potential as a mucosal vaccine candidate that could offer broad-range cross-protection against multiple strains of influenza virus. We focused on the advantages of adopting mucosal vaccination strategy, which has been shown to effectively target both systemic and mucosal immunity, over parenteral vaccination strategy [12,13,14]. Additionally, we compared the vaccination-induced immune responses generated following administration of our candidate vaccine virus via two different, intranasal and sublingual, mucosal routes.

## Materials and Methods

### Mice and Virus strain

Female BALB/c mice (5 week-old) were obtained from Orient Bio (Seoul, Korea). All of the mice were maintained under specific pathogen-free conditions in the experimental facility at the Ewha Womans University. The mouse-adapted influenza virus strains of A/Puerto Rico/8/34 (abbreviated PR8, H1N1) virus, A/California/04/09 (CA04, H1N1), A/Philippines/2/82 (A/Philippines, H3N2), and A/Vietnam/1203/04-PR8/CDC-RG-attenuated (A/Vietnam, H5N1) were used in this study for challenges. A/Vietnam/1203/04-PR8/CDC-RG-attenuated is a reassortant virus with the only HA genes of A/Vietnam/1203/04 (H5N1) origin in the genetic background of the high-growth strain A/Puerto Rico/8/34 (H1N1). Influenza virus stocks were grown in the embryonated chicken eggs. The allantoic fluid was collected and stored at -70°C.

### Cells

Human embryonic kidney 293 (HEK293) cells were grown in Dulbecco’s Modified Eagle Medium (DMEM) (Life Technologies, Gaithersburg, MD) supplemented with 10% fetal bovine serum. Madin-Darby Canine Kidney (MDCK) cells were grown in Minimal Essential Medium (MEM) with 10% fetal bovine serum.

### Construction of recombinant replication- defective adenoviruses

The viral RNA from PR8 virus was acquired by using QIAamp MinElute virus spin kit (Qiagen, Valencia, CA) in accordance with the manufacturer’s instructions. The cDNA corresponding to NP gene was generated by RT-PCR using forward primer (5’-GGGTACCGCCACCATGGCGTCCCAAGGCACC-3’) which contains a KpnI restriction enzyme site and the Kozak sequence to enhance translation and the reverse primer (5’-TTCTAGATTAATTGTCGTACTCCTC-3’) that contains a stop codon and an XbaI restriction enzyme site at 3’ terminus. The whole open reading frame was then digested with Kpn I/Xba I double digestion and inserted into the pShuttle-CMV vector. For generation of replication-defective adenovirus (serotype 5), the NP sequence was first inserted into adenovirus genome through homologous recombination as described previously [15]. Subsequently, recombinant adenoviral DNA containing the NP gene was transfected into HEK293 cells to generated rAd/NP virus. Mock adenovirus (rAd/Mock) was generated by the same method using the vacant pShuttle-CMV vector. The recombinant adenoviruses were amplified on HEK293 cells and purified by double CsCl_2_ density-gradient ultracentrifugation. The expression of NP protein by rAd/NP was confirmed by infecting HEK293 cells at multiplicity of infection (MOI) of 10 and immunoblotting using mouse polyclonal PR8-specific anti-serum and horseradish peroxidase (HRP)-conjugated goat anti-mouse IgG (Invitrogen, Eugene, OR) as a secondary antibody. To extract cellular proteins from the infected-HEK293 cells, cell lysates were prepared by resuspending cell pellets with a buffer containing 50 mM Tris-HCl, 150 mM sodium chloride, 1% NP-40, 0.5% sodium deoxycholate and 0.1% SDS, and clear supernatants and pellets were separated by centrifugation.

### Vaccination and Challenge

Female BALB/c mice were kept under specific pathogen-free conditions. For vaccination, 5 week-old mice were inoculated with varying doses of rAd/NP vaccine through intranasal (i.n.) or sublingual (s.l.) route. For i.n. immunization, mice were lightly anesthetized by isoflurane (Ifran®; Hana Pharm, Kyonggi-Do, Korea), and 1 × 10^7^ to 1 × 10^8^ plaque forming unit (PFU) of rAd/NP or 1 × 10^8^ PFU rAd/Mock in a volume of 50 µl of phosphate-buffered saline (PBS) was applied to the both nostrils. For s.l. immunization, mice were anesthetized by intraperitoneal injection of 100 mg/kg of body weight ketamine (Yuhan Co., Seoul, Korea) and 10 mg/kg of body weight xylazine hydrochloride (Bayer, Kyonggi-Do, Korea), as described elsewhere [16,17]. And then forceps were placed under the tongue of the mouse and its mouth was stretched open. The total volume of vaccines were kept to <5 µl to avoid swallowing effects. The s.l. groups were secondarily immunized by the same procedure two weeks after primary immunization. Three weeks after last immunization, mice were lightly anesthetized by isoflurane and challenged i.n. with 10 LD_50_ of PR8, CA04, A/Philippines or A/Vietnam. All animal studies were performed according to the guidelines of Ewha Womans University Institutional Animal Care and Use Committee (IACUC, Approval No. 2011-01-032).

### BAL and ELISA

At five days post challenge, subsets of mice were sacrificed and tracheotomy was executed. The lung airways were washed with 1 ml of PBS. The collected bronchoalveolar lavage (BAL) fluid was centrifuged and supernatants were used for measuring secretory IgA titers. Blood was acquired from the retro-orbital plexus by a heparinized capillary tube, centrifuged and serum collected was stored at -70°C. Antibody titers from immunized mice were measured by a direct enzyme-linked immunosorbent assay (ELISA). Briefly, for coating antigens, 1.8 × 10^7^ PFU of PR8 virus in allantoic fluid was disrupted with 0.5% Triton X-100. Next, 96-well plates were coated with 100 μl/well of split influenza virus diluted in PBS (1 : 500) and incubated overnight at 4°C, and then blocked with PBS containing 1% non-fat milk and 0.05% Tween 20 for 2 h at RT. Samples of sera or BAL fluids were added in serial dilutions and incubated for 2 h. After a washing step with PBS containing 0.05% Tween 20, HRP-conjugated goat anti-mouse IgG (Invitrogen) or HRP-conjugated goat anti-mouse IgA (Zymed Laboratories, San Francisco, CA) as a secondary antibody to measure NP-specific IgG in the sera or NP-specific IgA in the BAL fluids, respectively. For color development, 3,3’,5,5’-tetramethylbenzidine (KPL, Gaithersburg, MD) was added and stopped by adding 1 M H_3_PO_4_. The color development was analyzed at 450 nm by a Thermo Multiskan® EX (Vantaa, Finland).

### Lung virus titer measurements

Five days after influenza virus challenge, a subset of each group was euthanized and the influenza vial titers in the lungs were measured as described elsewhere [18]. Briefly, the lung tissues were removed into PBS and processed through a 70-µm cell strainer (BD Labware, Franklin Lakes, NJ) with 3 ml MEM. The supernatants were collected by centrifugation, and virus titers in the supernatants were analyzed by standard plaque assay on subconfluent MDCK cells. The data are expressed as the PFU per gram of lung tissue.

### Preparation of lymphocytes and flow cytometric analysis

The lungs perfused with 5 ml PBS including 10 U/ml heparin (Sigma, St. Louis, MO) using a syringe with 25-gauge needle through the right ventricle were dissected and collected. To obtain single-cell suspensions, the tissues were homogenized with 3 ml Iscove’s Modified Dulbecco’s Medium (IMDM) through 70-µm cell strainers. Following centrifugation, lymphocytes were resuspended in fresh IMDM and erythrocytes were removed by red blood cell lysing buffer (Sigma). After washing with IMDM, cells were washed two times with FACS buffer (0.5% FBS, 0.09% NaN_3_ in PBS) and were blocked with purified rat anti-mouse CD16/CD32 (BD Pharmingen, San Diego, CA) and 5 µg/ml streptavidin (Invitrogen). Then, cells were stained with anti-mouse CD8a-APC (clone 53-6.7; Biolegend, San Diego, CA), anti-mouse CD44-FITC (clone IM7; Biolegend) and K^d^/NP_147-155_(TYQRTRALV)-tetramer-PE. After staining, the cells were fixed in PBS-2% (wt/vol) paraformaldehyde and analyzed using FACSCalibur flow cytometer (BD Biosciences, San Diego, CA) and Flowjo software (TreeStar Inc., Ashland, OR).

### Statistical methods

All data were plotted as mean±standard error (n=5) and the difference comparison was conducted by using an unpaired, two-tailored Student t-test. The difference was considered statistically significant when P values were ≤ 0.05.

## Results

### A. Generation and characterization of recombinant adenovirus expressing NP of influenza virus

**Figure 1:**
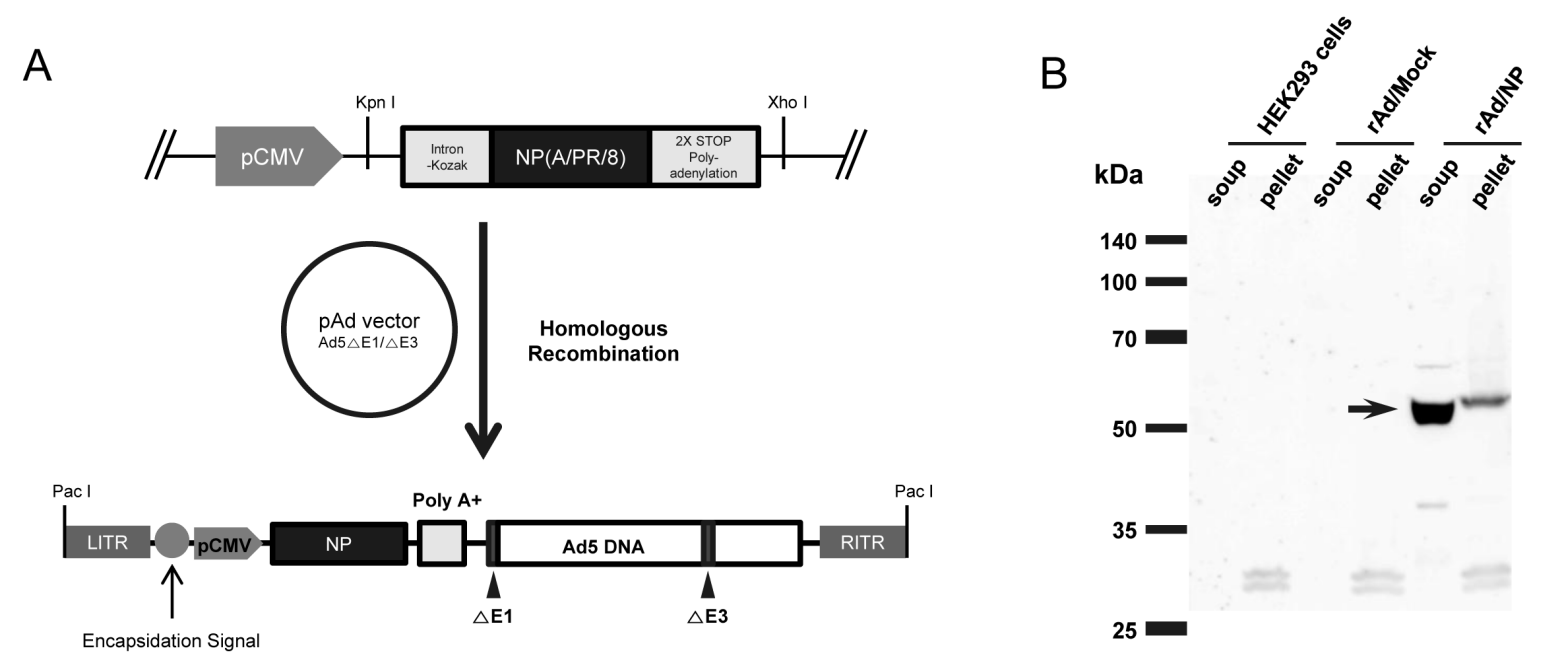
Generation of replication-defective adenovirus expressing NP gene of influenza virus (rAd/NP). (A) A schematic diagram of rAd/NP recombinant genome generated after homologous recombination between the shuttle vector and adenoviral genome. A shuttle vector plasmid containing NP gene of PR8 virus was constructed and recombined with the pAd-Easy vector (Ad5-△E1/E3). (B) Expression of NP in the lysates of HEK293 cells infected with rAd/NP. The expression of NP (indicated by the arrow) was confirmed by immunoblotting assay as described in the Materials and Methods. B. Humoral immune responses induced by mucosal rAd/NP immunization.

The full length coding region of NP gene from PR8 under the control of CMV promoter followed by a Kozak sequence, which enhance the translation of gene inserts, and polyadenylation stop signal (polyA) was inserted into early region 1 (E1) of the adenovirus genome by homologous recombination, resulting in the generation of recombinant replication-defective adenovirus expressing PR8-dervied NP (rAd/NP) (Figure 1A). The proper expression of NP in our recombinant adenovirus was evaluated by infecting HEK293 cells with rAd/NP and conducting immunoblot analysis with the infected cell lysates using PR8-specific polyclonal antibody. We detected a robust, single band at approximate molecular weight of 56 kDa representing NP in rAd/NP-infected HEK293 cell lysates. However, no such band was detected in uninfected HEK293 cell lysates or rAd/Mock-infected HEK293 cell lysates that were used as negative controls (Figure 1B).

**Figure 2:**
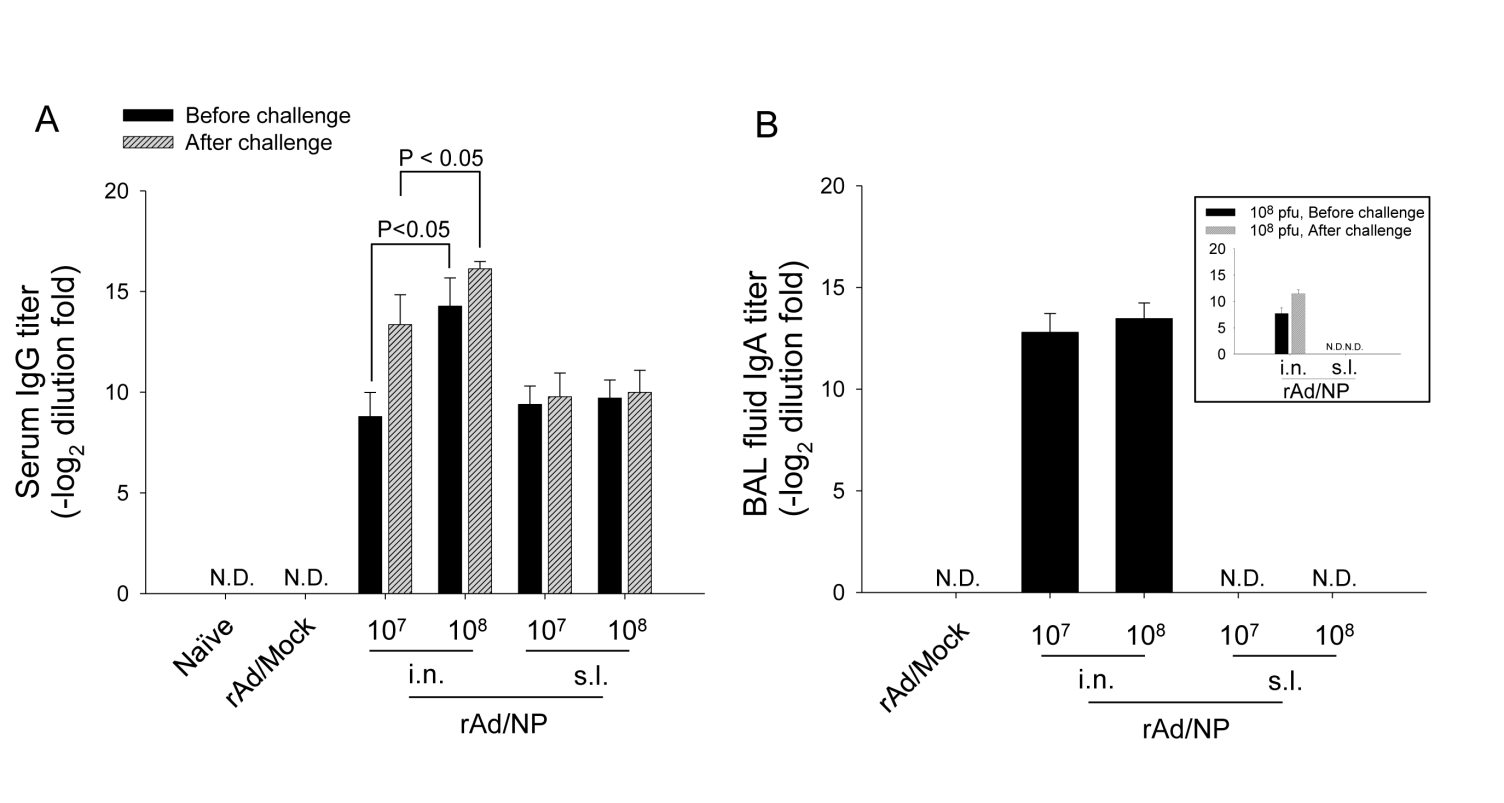
Characterization of humoral immune responses induced by mucosal rAd/NP vaccination. BALB/c mice were immunized intranasally or sublingually with 1×10^7^ or 1×10^8^ PFU of rAd/NP. Two weeks after the primary immunization, animals that previously received rAd/NP via sublingual route were boosted with the same dose of rAd/NP used during the primary immunization. Control mice were immunized intranasally with 10^8^ PFU of rAd/Mock or with equal volume of PBS. (A) Systemic PR8-specific IgG antibody titers were measured by ELISA in sera harvested three weeks after their last immunization regimen and subsequent challenge with 10 LD_50_ of PR8 virus at two different time points: pre-challenge and post-challenge. (B) Mucosal PR8-specific IgA titers were determined by ELISA in BAL from mice immunized i.n. or s.l. with rAd/NP and subsequent challenge with 10 LD_50_ of PR8 virus at two different time points: pre-challenge and post-challenge. The results represent Log_2_ endpoint values averaged from five individual mice.

In order to determine the optimal route and dose of rAd/NP vaccination to elicit appropriate influenza virus NP-specific immune responses, BALB/c mice were inoculated intranasally or sublingually with either 1×10^7^ or 1×10^8^ PFU of rAd/NP. Also, mice were immunized intranasally with 1×10^8^ PFU of rAd/Mock to be used as negative control. In our preliminary studies, a single s.l. immunization with rAd/NP was insufficient to produce detectable immunogen-specific antibody levels in BALB/c mice (data not shown). Therefore, in order to enhance the efficacy of immunogen-specific antibody responses for the s.l. vaccination, sublingually primed mice were boosted two weeks after their first immunization using the same vaccination scheme used during their priming. However, all mice immunized intranasally received a single immunization with the indicated dose of rAd/NP or rAd/Mock. Subsequently, three weeks after their respective final immunization, sera were harvested from all immunized mice, and the levels of PR8-specific IgG in the immune sera were determined via ELISA using detergent-disrupted PR8 virus as the coating antigen. All i.n. and s.l. vaccination groups that received rAd/NP elicited significant levels of PR8-specific serum IgG compared to control mice given rAd/Mock (Figure 2A). Interestingly, while mice immunized via s.l. route generated comparable levels of PR8-specific serum IgG titers regardless of the administered vaccine dose, mice immunized via i.n. route showed significant dose-dependent differences in serum IgG titers between the two vaccination doses used. Further, when PR8-specific serum IgG levels were determined in the sera harvested from PR8-infected mice at day 5 post-challenge, we observed significant increase in the PR8-specific IgG titers in mice vaccinated intranasally with either dose of rAd/NP. However, such increase in PR8-specific serum IgG titers following PR8-challenge was not observed in sublingually vaccinated mice. In summary, these data indicate that both i.n. and s.l. immunizations with rAd/NP elicit considerable levels of PR8 NP-specific serum IgGs and that PR8-challenge following rAd/NP immunization significantly increases PR8-specific serum IgG levels, compared to that of pre-challenge levels, in intranasally immunized mice, but not in sublingually immunized mice.

Additionally, when the levels of NP-specific mucosal IgA in BAL collected at day 5 post-PR8-challenge were evaluated, we observed that extensive levels of PR8-specific IgA titers were detected in mice vaccinated intranasally with rAd/NP regardless of the administered vaccine dose. We also observed that PR8-challenge following 1×10^8^ PFU of rAd/NP immunization increases PR8-specific mucosal IgA levels, compared to that of pre-challenge levels, in intranasally immunized mice. As expected, BAL fluid collected from mice immunized with rAd/Mock contained no detectable levels of PR8-specific mucosal IgA. Interestingly, however, we did not detect PR8-specific mucosal IgAs in BAL collected from mice that were given rAd/NP via s.l. route (Figure 2B). Taken together, these results suggest that rAd vector-based vaccine expressing the NP gene of PR8 virus is capable of eliciting NP-specific immune responses and that a single immunization with rAd/NP via i.n. route sufficiently induces strong systemic as well as mucosal immunity to PR8 virus as represented by increased serum IgG and mucosal IgA levels, respectively.

### C. Cytotoxic T-lymphocyte responses induced by mucosal rAd/NP immunization

**Figure 3:**
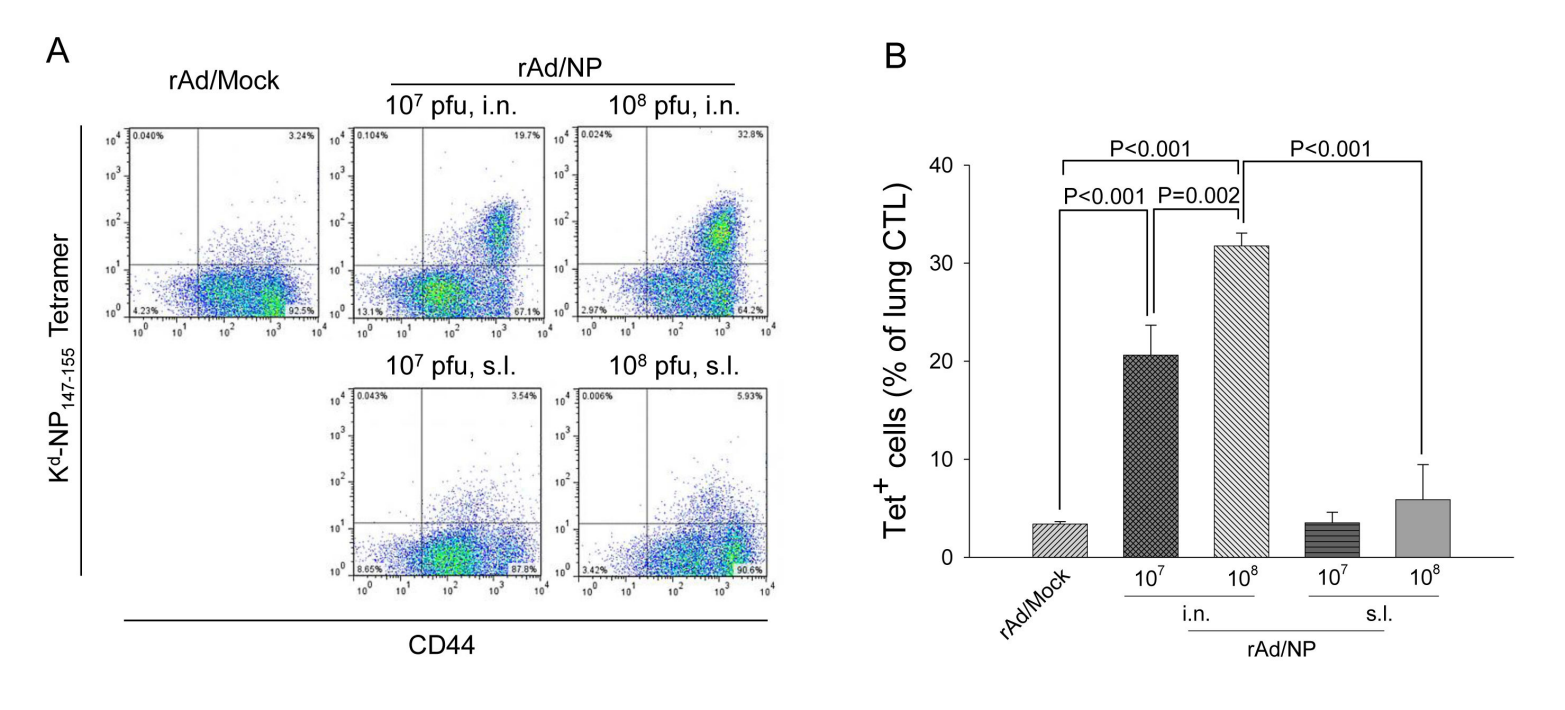
Analysis of influenza virus NP-specific CD8 T-cell responses in the lungs of rAd/NP-immune mice. BALB/c mice were immunized intranasally or sublingually with 1×10^7^ PFU or 1×10^8^ PFU of rAd/NP. Control mice were immunized intranasally with rAd/Mock. Mice that received rAd/NP via sublingual route were boosted two weeks after the primary vaccination. Subsequently mice in all vaccination groups were challenged intranasally with 10 LD_50_ of PR8 virus three weeks after their last immunization. (A) At day 5 post-challenge, lung cells were harvested from mice (n = 5) in each group and stained with K ^d^/NP_147-155_ tetramer, anti-CD8, and anti-CD44 antibody. The percentage of K ^d^/NP_147-155_ tetramer-positive, CD44-positive cells among total lung CD8-positive cell population was indicated in the upper right quadrant. (B) The average percentages of K^d^/NP_147-155_ tetramer-specific CD8 T cells in the lungs of five mice per group.

Previous studies have reported that influenza virus NP contains a K^d^-restricted immunodominant CD8 T-cell epitope between amino acids from 147 to 155, against which dominant CD8 T-cell responses are elicited during influenza virus infection. In order to determine the ability of rAd/NP vaccination to induce CD8 T-cell responses against the mentioned epitope, the levels of K^d^/NP_147-155_ tetramer-specific CD8 T-cell recruitment in the lungs following rAd/NP vaccination and subsequent PR8-challenge were evaluated. Briefly, mice were immunized i.n. once with either 1×10^7^ or 1×10^8^ PFU of rAd/NP or immunized s.l. with rAd/NP using the same prime-and-boost vaccination regimen as aforementioned. Again, a group of mice immunized i.n. with rAd/Mock was used as our negative control. All immunized mice were then challenged with a lethal dose of PR8. At day 5 post-challenge, mice lungs were harvested and K^d^/NP_147-155_ tetramer-specific CD8 T-cell frequency was determined via flow cytometry. As expected, we observed significant increase in the percentage (of total lung CD8 T cells) of K^d^/NP_147-155_ tetramer-specific CD8 T cells in the lungs of mice that received rAd/NP via i.n. route compare to the lungs of control mice that received rAd/Mock (Figure 3). Moreover, this increase in NP-specific CD8 T-cell recruitment was dose-dependent, as higher percentage (of total lung CD8 T cells) of NP-specific CD8 T cells were detected in mice that received 1×10^8^ PFU dose compared to those that received 1×10^7^ PFU (Figure 3). However, we did not observed any significant differences in the levels of K^d^/NP_147-155_ tetramer-specific CD8 T cells in any of the s.l. immunized groups compared to rAd/Mock immunized control group, notwithstanding the dose of rAd/NP vaccine administered (Figure 3). Such phenomena should be underscored as similar trend was observed in the PR8-specific serum IgG and mucosal IgA levels between the animals that received differential doses of rAd/NP vaccine via i.n. route.

### D. Protective efficacy of mucosal rAd/NP immunization against homologous influenza virus challenge

**Figure 4:**
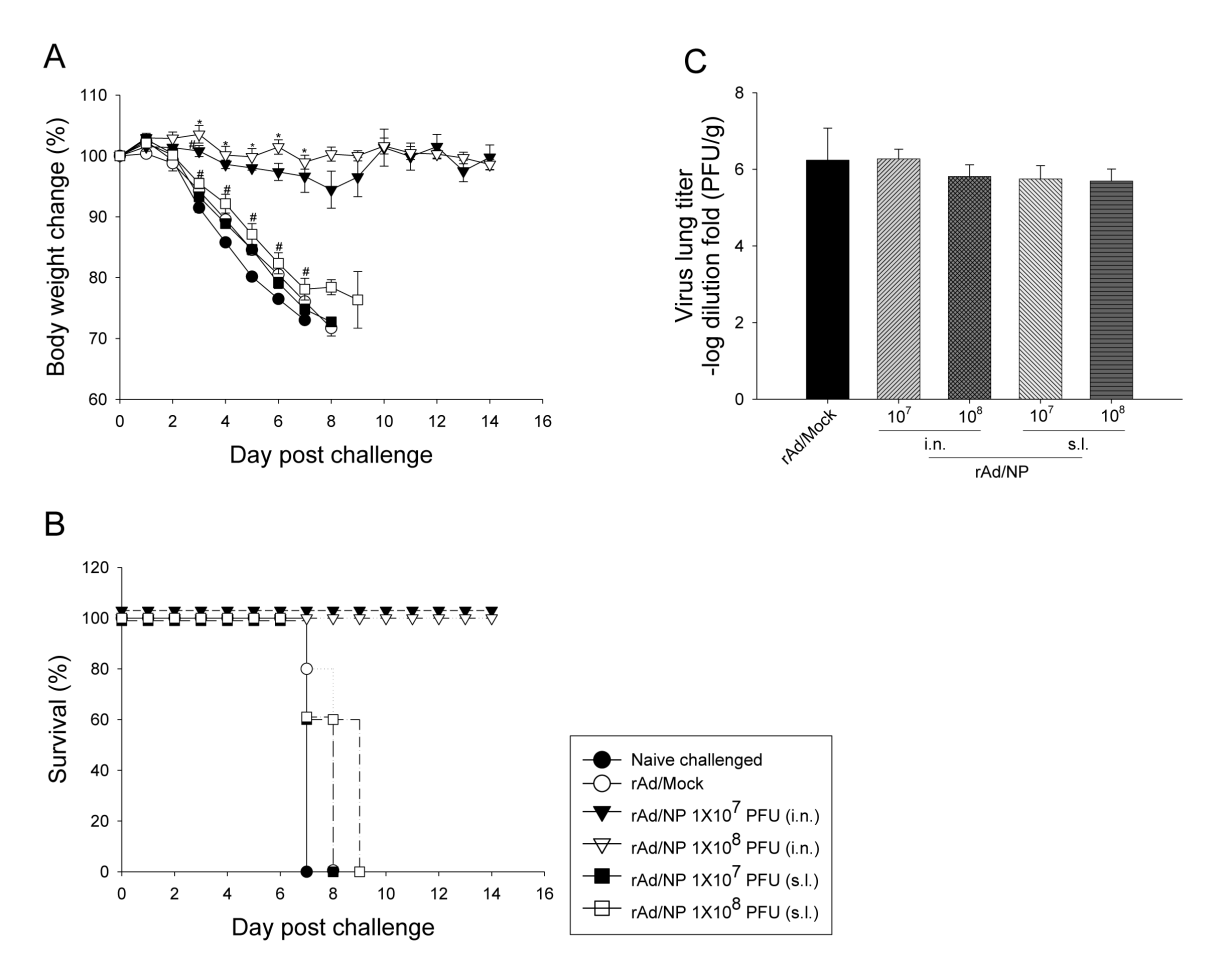
rAd/NP immunization protects mice from weight loss and mortality following homologous PR8 challenge. BALB/c mice were immunized intranasally or sublingually with 1×10^7^ PFU or 1×10^8^ PFU of rAd/NP. Control mice were immunized intranasally with rAd/Mock. Mice that received rAd/NP via sublingual route were boosted two weeks after the primary vaccination. Subsequently mice in all vaccination groups were challenged intranasally with 10 LD_50_ of PR8 virus three weeks after their last immunization. (A) Body weight and (B) survival rate were recorded daily. (C) Viral replication levels were determined by plaque assay on MDCK cells in supernatant of lung homogenates harvested on day 5 post-challenge. Bars show log_10_ geometric mean titer ± SEM of five mice per group.

In order to determine whether rAd/NP immunization confer protection against homologous influenza virus infection, mice were challenge with 10 LD_50_ of live PR8 virus, three weeks after their last respective immunization regimens. Our data show that all groups that received i.n. immunization of rAd/NP, regardless of the administered vaccination dose, survived the lethal PR8 challenge and demonstrated considerable resistance to weight loss (Figure 4A and B). However, all mice that received either dose of rAd/NP via s.l. route as well as control mice that received rAd/Mock or un-immunized naïve mice succumbed to PR8 infection by day 9 post-challenge (Figure 4A and B). Interestingly, however, lung virus titers at day 5 post-challenge were detected at similar levels in all immunization groups (Figure 4C). It is probable that rAd/NP may induce long-lasting innate immunity that contributes complementarily with other specific immune arms to the control of the disease by uncharacterized mechanisms [19]. As a result, the protection may not necessarily correlate with virus titers detected in the lungs upon lethal challenge. Overall, these results indicate that i.n. immunization of rAd/NP can confer complete protection against the lethal homologous virus challenge while allowing competent virus replication to perpetuate even to day 5 post-challenge.

### E. Cross-protection against heterologous and heterosubtypic influenza virus challenge by mucosal rAd/NP immunization

**Figure 5:**
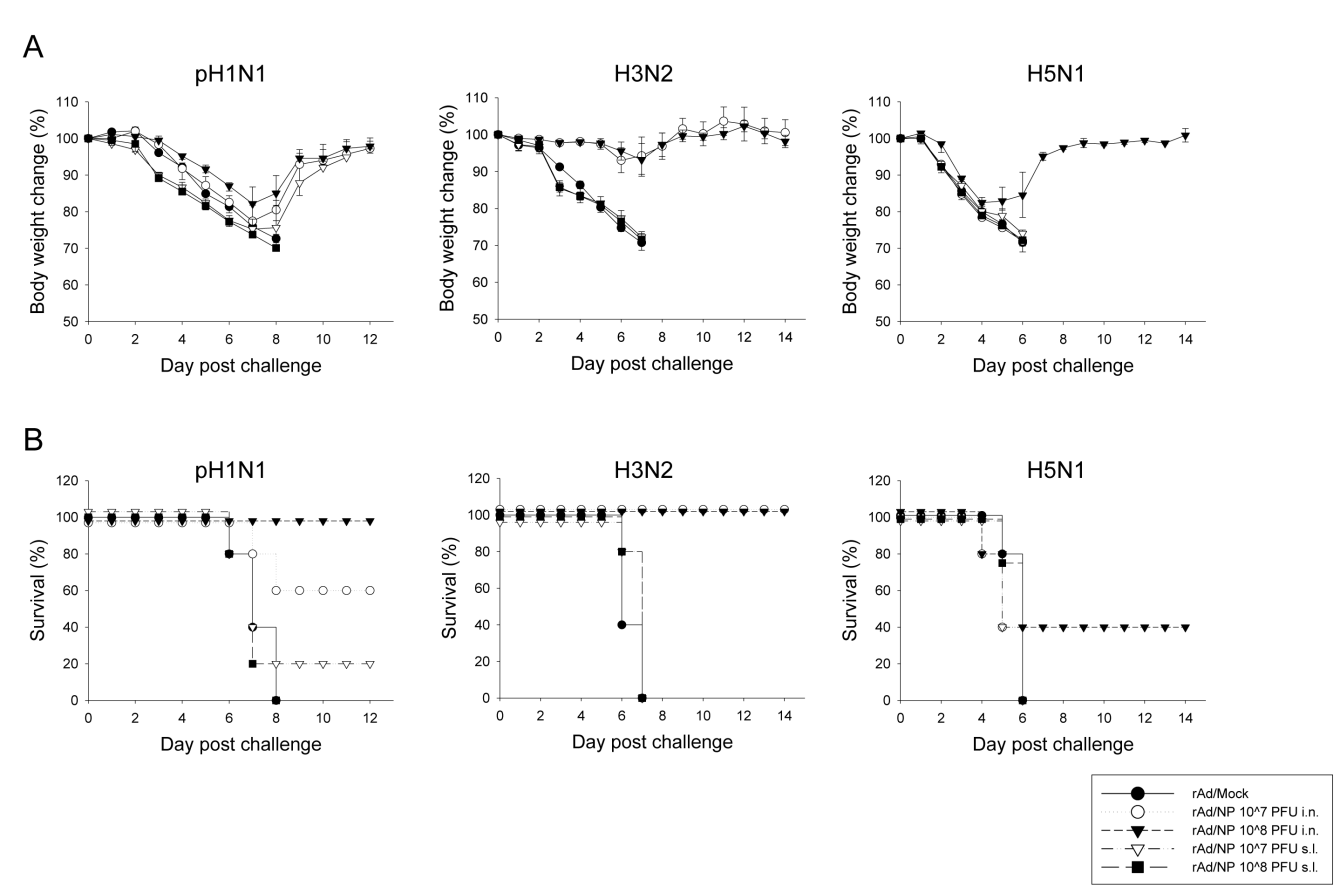
Analysis of protective efficacy against heterologous and heterosubtypic influenza virus challenge by mucosal rAd/NP vaccination. BALB/c mice were immunized intranasally or sublingually with 1×10^7^ PFU or 1×10^8^ PFU of rAd/NP. Control mice were immunized intranasally with rAd/Mock. Mice that received rAd/NP via sublingual route were boosted two weeks after the primary vaccination. Subsequently mice in all vaccination groups were challenged with 10 LD_50_ of CA04 (H1N1) virus, A/Philippines (H3N2) virus, or A/Vietnam (H5N1) virus. (A) Body weight and (B) survival rate were recorded daily. Error bars in weight loss graph indicated mean ± SEM of five mice per group.

In order to determine whether immunization with rAd/NP can offer cross-protection against heterologous and/or heterosubtypic influenza infections [20], immune mice were challenged at 3 weeks after their last respective immunization regimen. First, to investigate the protective efficacy of rAd/NP immunization against heterologous influenza virus infection but with the same H1N1 subtype, mice were challenged with a lethal dose of CA04 and monitored daily for body weight change and mortality. The group of mice that received i.n. immunization of 1×10^8^ PFU of rAd/NP experienced some weight loss, but 100% of the animals survived the lethal challenge. The group that received i.n. immunization of 1×10^7^ PFU of rAd/NP experienced somewhat heavier weight loss compared to the group that received the higher dose, and 60% of the animals survived the challenge (Figure 5). However, all animals that received s.l. immunization experienced substantial weight loss. Interestingly, we observed that 20% of the animals that received 1×10^7^ PFU of the vaccine virus via s.l. route survived the lethal challenge whereas all animals that received 1×10^8^ PFU of the vaccine virus via the same route succumbed to the challenge.

Next, in order to evaluate heterosubtypic protection offered by rAd/NP vaccination, mice were challenged with a lethal dose of influenza A/Philippines (H3N2) or A/Vietnam (H5N1) and monitored daily for body weight change and mortality. All mice immunized with rAd/NP via s.l. route, notwithstanding the administered vaccine dose, suffered considerable weight loss before succumbing to death within 8 days following A/Philippines influenza virus challenge (Figure 5). In contrast, both groups of i.n. immunized animals that received either 1×10^7^ or 1×10^8^ PFU of the vaccine were completely protected and experienced minimal weight loss following A/Philippines challenge (Figure 5).

Overall, influenza A/Vietnam challenge produced steeper weight loss in animals compared to other influenza virus challenges. Both s.l. immunized groups that received either 1×10^7^ or 1×10^8^ PFU of the vaccine suffered quick and severe weight loss upon H5N1 challenge and, thereafter, succumbed to death within 6 days (Figure 5). The group that received 1×10^7^ PFU of rAd/NP via i.n. route also experienced severe weight loss comparable to s.l. immunized groups and were not protected from the lethal challenge. Interestingly, however, i.n. immunization with 1×10^8^ PFU of rAd/NP provided 40% of protection against the challenge with the same dose of H5N1 virus (Figure 5B). Taken together, our data demonstrate that rAd/NP immunization via i.n. route, but not s.l. route, provides complete protection against heterologous H1N1 and heterosubtypic H3N2 virus challenges and suggest that a high dose intranasal immunization may be required to confer protection against H5N1 virus challenge.

### F. T cell responses after heterosubtypic influenza virus challenge

**Figure 6:**
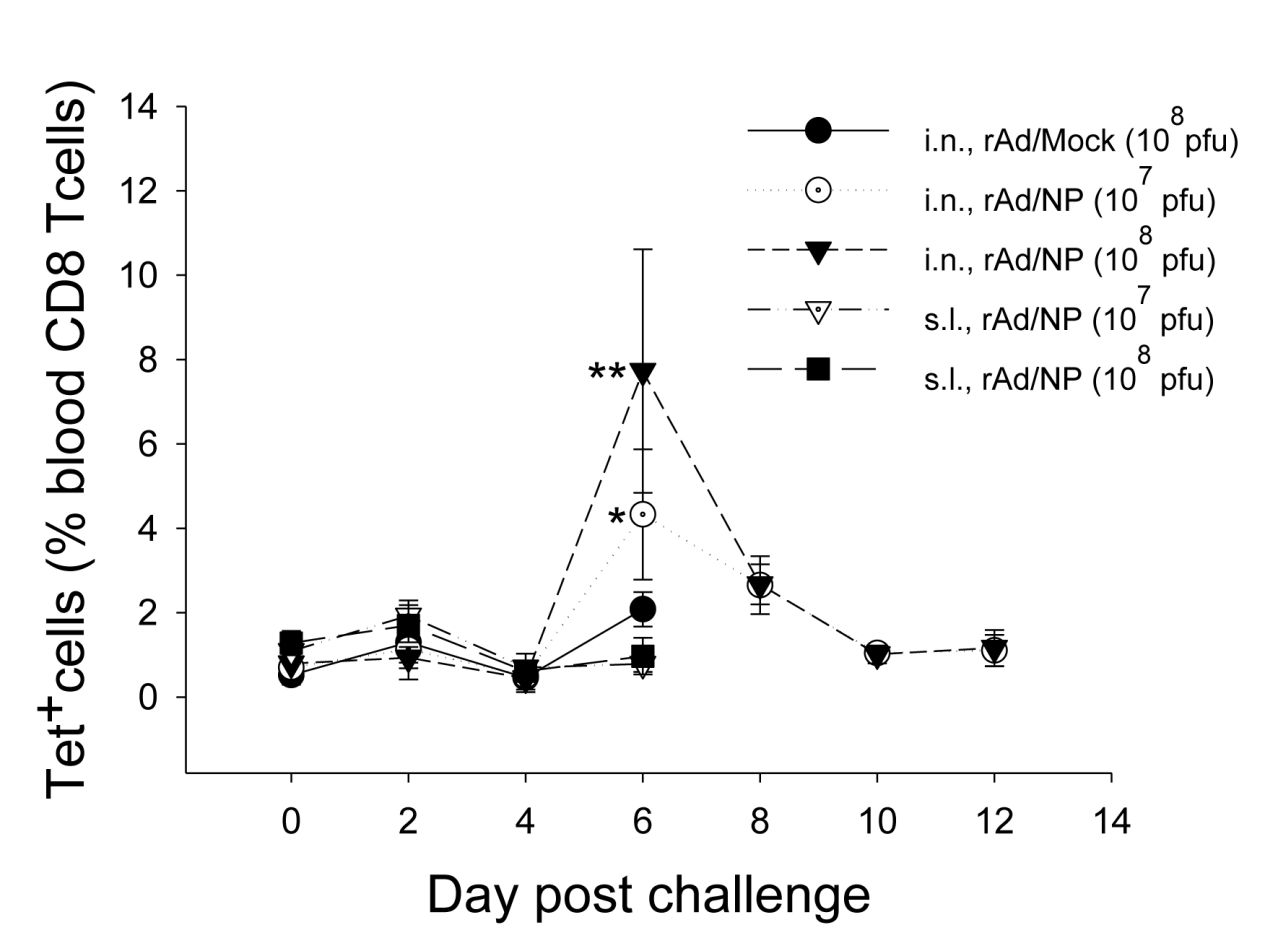
Analysis of NP-specific CD8 T cells in the peripheral blood after rAd/NP immunization and heterosubtypic influenza virus challenge. The BALB/c mice immunized with 1×10^7^ or 1×10^8^ PFU of rAd/NP were challenged with 10 LD_50_ of A/Philippines (H3N2). At each indicated time point after influenza virus challenge, peripheral blood leukocytes from surviving mice of each group were isolated and stained as described in the Materials and Methods. The proportions of NP-specific CD8 T cells were measured by K^d^/NP_147-155_ tetramer staining and flow cytometric analysis. Error bars in the percentages indicate mean ± SEM (n=2~5). Statistical significance with rAd/Mock (*, p=0.03; **, p=0.008).

Complete protection offered by i.n. immunization of rAd/NP during heterologous H1N1 or heterosubtypic H3N2 virus challenge rendered us to investigate whether the observed cross-protection correlates with the magnitude NP-specific CD8 T-cell responses, given that influenza NP contains a conserved immunodominant CD8 T-cell epitope as indicated previously. In order to determine the possible presence of such correlation, blood lymphocytes of mice challenged with 10 LD_50_ dose of influenza A/Philippines were analyzed by K^d^/NP_147-155_-tetramer staining from day 0 to day 12 post-challenge. As expected, all animals that received control rAd/Mock immunization or rAd/NP immunization via s.l. route succumbed to death by day 7 post-challenge (Figure 5). However, i.n. immunization with rAd/NP, notwithstanding the administered dose, conferred complete protection (Figure 5). Moreover, at day 6 post-challenge, significant increases in the percentage of NP tetramer-positive blood CD8 T lymphocytes were observed in mice immunized via i.n. route, and greater proportion of NP-specific blood CD8 T lymphocytes was observed in mice that received i.n. immunization of 1×10^8^ PFU dose of rAd/NP than in mice that received 1×10^7^ PFU dose (Figure 6). Taken together, our results strongly suggest that NP-specific CD8 T cells, primed by i.n. rAd/NP immunization, can be successfully recalled during a subsequent heterosubtypic influenza virus infection and can be ascribed to cross-protection observed in previous figures. 

## Discussion

Recent abrupt outbreak of influenza pandemic caused by a new swine/human/avian-origin influenza A (H1N1) virus raised a significant concern for global public health and awareness for the necessity of better preparedness against potential recurrence of influenza pandemic. Currently, inactivated and live-attenuated influenza vaccines are widely used for vaccination in humans. However, the efficacy of these vaccines largely depends on the antigenic relatedness of the vaccine virus to the circulating influenza virus strains. Given that influenza virus is a highly dynamic virus that readily undergoes genetic shifts and drifts, development of vaccines that offer broad coverage against different strains and subtypes of influenza virus is imperative.

Previous studies have identified influenza A virus NP as a major target antigen for cross-reactive influenza virus-specific CD8 T cells and suggested that strategy to induce NP-specific CD8 T cells should be considered for the development of broadly protective influenza virus vaccines [21,22,23,24]. Accordingly, we engineered a recombinant adenovirus expressing full-length NP derived from PR8 influenza virus as a potential vaccine candidate in order to investigate whether priming for NP-specific immune responses could offer cross-protection against different strains and subtypes of influenza virus. However, the use of adenovirus-based vaccine raises an important concern regarding development of vector-specific immunity in vaccine recipients as pre-existing vector immunity could interfere with the vaccine efficacy in scenarios requiring multiple booster vaccinations [25]. To address this challenge, we chose to administer of our recombinant adenovirus vaccine via mucosal route as previous studies have shown that a single immunization via nasal route can bypass the development of vector-specific immunity to adenovirus vector in human while eliciting potent immune responses specific for the vaccine antigen [26,27,28]. Moreover, the airway mucosa is the main entry point for various invading pathogens, functioning as the first line of defense against respiratory infections [29]. The mucosal immune system is distinct from the systemic immune system as it possesses distinctly organized immunological tissues of its own which function to maintain the homeostasis within the mucosa [30]. Current method of delivering influenza vaccines via parenteral route relies on the systemic induction of virus-specific IgGs for protection. However, previous studies have reported that influenza vaccination efficacy is also closely associated with the immune responses induced within the respiratory mucosa [31,32,33]. Given that parenteral influenza vaccines presently in use are inefficient in stimulating immune responses in mucosal tissues [34], our study examined the potential application of mucosal immunization strategy which has been shown to effectively target both systemic and mucosal immunity [12,13,14]. However, there is a safety issue concerning the redirection of antigens to the central nervous system in i.n. immunization [35], while s.l. route is thought to be relatively safe [36]. Therefore, in our present study, we assessed i.n. and s.l. immunizations as an approach to evaluate the protective efficacy generated by mucosal vaccination of our rAd-based influenza vaccine, as these two routes of immunization have been shown to promote induction of protective immune responses characterized by the localized responses in the respiratory mucosa [37,38] as wells as systemic induction of vaccine antigen-specific responses [39]. Additional aspects of our study focused on the comparison of humoral and cell-mediated immune responses generated following administration of our vaccine virus via these two different mucosal routes. Further, we wanted to determine the specific branches of immunity, primed by mucosal rAd/NP immunization, which correlate to the establishment cross-protection against different influenza virus strains.

In our present study, we demonstrated that rAd/NP immunization increases the frequency of NP-specific CD8 T cells recruited to the lungs of i.n. immunized mice following homologous challenge with PR8 virus. Accordingly, complete protection against PR8 challenge was observed only in the groups that received the vaccine virus via i.n. route, indicating that NP-specific CTL response may be directly correlated to protection against homologous influenza virus infection. Moreover, protection against heterosubtypic influenza virus infection may also be correlated to NP-specific CTL response as NP-specific blood CD8 T lymphocyte levels considerably increased (starting from day 4 post-challenge) in mice that survived the lethal challenge with heterosubtypic H3N2 virus. However, i.n. immunization failed to confer complete protection against H5N1 infection as only 40% of mice in the group that were given 1×10^8^ PFU dose of rAd/NP survive this lethal challenge. Our explanation for such lack of protection against H5N1 virus is that, although the immunodominant CD8 T-cell epitope within in NP (NP_147-155_) are fully conserved among all influenza A subtypes, the magnitude and characteristic of CTL response elicited during differential influenza virus infection may be distinct for each influenza virus strain causing the concurrent infection. Hence, even subtle differences in the CTL responses may affect the degree of protection offered during influenza infection caused by different influenza virus strains [40,41]. Mucosal immunization of mice with rAd/NP also dramatically increases NP-specific IgG levels in the serum independent of the immunization route. However, we observed that subsequent challenge with the homologous influenza virus additionally increases the NP-specific serum IgG levels in i.n. immunized mice, but not in s.l. immunized mice. Further, substantial levels of NP-specific respiratory mucosal IgAs were detected in i.n. immunized mice, whereas no such IgAs were detected in s.l. immunized mice. Thus, it is possible that the presence of influenza NP-specific IgGs and IgAs in the respiratory mucosa may also be involved in the protection against the lethal influenza challenges [42,43], even though the exact mechanisms remain to be determined further. The immunization with adenovirus vector encoding NP induced both cellular and antibody responses. It has been shown recently that influenza virus-infected cells can be eliminated by anti-M2e IgG-mediated cellular cytotoxicity or phagocytosis since these cells express M2 on their surface after infection [44]. Similarly, the NP-specific antibodies may interact with the viral NP expressed on cell surface of infected cells and mediate cell lysis by antibody-dependent cellular cytotoxicity.

Overall, our study demonstrates that a single i.n. administration of rAd/NP confer cross-protection against lethal challenge by different influenza virus strains, while s.l. administration of the same vaccine failed to confer protection, and we ascribe the high levels of NP-specific CTLs and antibodies found in intranasally immunized mice to the observed cross-protection. Given that influenza NP contains a conserved immunodominant CD8 T-cell epitope shared among all influenza A virus and that mucosal immunization can stimulate both mucosal and systemic immune responses, we believe that i.n. immunization with rAd/NP can induce protective immunity against different strains of influenza virus by priming for cross-reactive NP-specific CTL response and possibly by local and systemic induction of NP-specific antibodies. As such, our vaccination approach, examined in the present study, could be further explored as a next-generation influenza vaccination strategy which could generate broadly protective immunity against multiple influenza virus strains and, thus, greatly reduce influenza virus-related public health burden.
