# Correction: Mucosal Vaccination with Recombinant Adenovirus Encoding Nucleoprotein Provides Potent Protection against Influenza Virus Infection

**DOI:** 10.1371/annotation/c2c0b5cc-ce3e-4c1f-a821-caed5697d72d

**Published:** 2013-11-15

**Authors:** So-Hee Kim, Joo Young Kim, Youngjoo Choi, Huan H. Nguyen, Man Ki Song, Jun Chang

The legend for Figure 1 is incorrect. The legend should read: "(A) A schematic diagram of rAd/NP recombinant genome generated after homologous recombination between the shuttle vector and adenoviral genome. A shuttle vector plasmid containing NP gene of PR8 virus was constructed and recombined with the pAd-Easy vector (Ad5-△E1/E3). (B) Humoral immune responses induced by mucosal rAd/NP immunization. Expression of NP in the lysates of HEK293 cells infected with rAd/NP. The expression of NP (indicated by the arrow) was confirmed by immunoblotting assay as described in the Materials and Methods. doi:10.1371/journal.pone.0075460.g001." The title of Figure 1 is correct as it is.

In addition, in the Author Contributions Statement, the third and fourth authors (YC and HHN) were incorrectly omitted from the list of authors who wrote the manuscript.

